# Misoprostol use in obstetrics: Number 6 – June 2023

**DOI:** 10.1055/s-0043-1770931

**Published:** 2023-07-21

**Authors:** Coordinator, Roseli Mieko Yamamoto Nomura, National Specialized Commission on Maternal Mortality, Marcos Nakamura-Pereira, National Specialized Commission on Fetal Medicine, Roseli Mieko Yamamoto Nomura, Maria de Lourdes Brizot, National Specialized Commission on Childbirth, Puerperium and Abortion Care, Alberto Trapani Júnior, National Specialized Commission on Sexual Violence and Pregnancy Interruption Provided for by Law, Helena Borges Martins da Silva Paro, Cristião Fernando Rosas, Robinson Dias de Medeiros, National Specialized Commission on Antenatal Care, Fernanda Garanhani Surita, Adriana Gomes Luz, National Specialized Commission on High-Risk Pregnancy, Rosiane Mattar, Elton Carlos Ferreira, National Specialized Commission on Urgency and Emergency, Álvaro Luiz Lage Alves, Eduardo Cordioli, National Specialized Commission on Professional Defense and Appreciation, Lia Cruz Vaz da Costa Damásio, Maria Celeste Osürio Wender, Revision, Antonio Braga

**Affiliations:** 1 Escola Paulista de Medicina, Universidade Federal de São Paulo, SP, Brasil; 2 Instituto Fernandes Figueira, Rio de Janeiro, RJ, Brasil; 3 Escola Paulista de Medicina, Universidade Federal de São Paulo, SP, Brasil; 4 Faculdade de Medicina, Universidade de São Paulo, São Paulo, SP, Brasil; 5 Departamento de Ginecologia e Obstetrícia, Universidade Federal de Santa Catarina, Florianüpolis, SC, Brasil; 6 Faculdade de Medicina, Universidade Federal de Uberlândia, Uberlândia, MG, Brasil; 7 Hospital Maternidade Escola de Vila Nova Cachoeirinha, São Paulo, SP, Brasil; 8 Universidade Federal do Rio Grande do Norte, Natal, RN, Brasil; 9 Universidade Estadual de Campinas, Campinas, SP, Brasil; 10 Universidade Estadual de Campinas, Campinas, SP, Brasil; 11 Escola Paulista de Medicina, Universidade Federal de São Paulo, SP, Brasil; 12 Universidade Estadual de Campinas, Campinas, SP, Brasil; 13 Hospital das Clínicas, Universidade Federal de Minas Gerais, Belo Horizonte, MG, Brasil; 14 Grupo Santa Joana, São Paulo, SP, Brasil; 15 Universidade Federal do Piaui, Teresina, PI, Brasil; 16 Universidade Federal do Rio Grande do Sul, Porto Alegre, RS, Brasil; 17 Universidade Federal do Rio de Janeiro, Rio de Janeiro, RJ, Brasil; Universidade Federal Fluminense, Niterüi, RJ, Brasil

## Key points

- Misoprostol is a prostaglandin E1 (PGE1) analogue that has been on the World Health Organization (WHO) List of Essential Medicines since 2005.
- Brazil has one of the most restrictive regulations in the world related to the use of misoprostol establishing it is exclusively for hospital use with special control, and sale, purchase and advertising prohibited by law.
- Misoprostol is currently the reference drug for pharmacological treatment in cases of induced abortion, both in the first trimester of pregnancy and at more advanced gestational ages.
- Misoprostol is an effective medication for cervical ripening and labor induction.
- Misoprostol is an essential drug for the management of postpartum hemorrhage.

## Recommendations

- The use of misoprostol is recommended for the following situations: legal abortion, uterine evacuation due to embryonic or fetal death, cervical ripening before labor induction (uterine cervix maturation), labor induction and management of postpartum hemorrhage.
- Misoprostol 800 mcg vaginally (four 200 mcg pills) is recommended for uterine evacuation in pregnancy loss up to 13 weeks.
- In cervical preparation for surgical abortion at less than 13 weeks of pregnancy, the use of misoprostol 400 mcg vaginally 3-4 hours before the procedure is recommended.
- The use of misoprostol alone according to the gestational age for uterine evacuation is recommended for termination of pregnancy in legal abortion.
- The use of vaginal misoprostol according to the gestational age is recommended for uterine evacuation in case of fetal death: at 13-26 weeks, 200 mcg every 4-6 hours; at 27-28 weeks, 100 mcg every 4-6 hours; and over 28 weeks, 25 mcg every six hours.
- The use of misoprostol at an initial dose of 25 mcg vaginally every 4-6 hours is recommended for cervical ripening and induction of labor with a live fetus in pregnancies over 26 weeks.
- The use of misoprostol for cervical ripening and induction of labor with a live fetus is not recommended in women with a previous cesarean section due to the greater risk of uterine rupture.
- Misoprostol is a safe and effective option for women with premature rupture of membranes and unfavorable uterine cervix, as long as they do not have contraindications for taking the medication, for example, previous cesarean section.
- Rectal misoprostol 800 mcg is recommended as part of the drug treatment of postpartum hemorrhage.
- In Brazil, misoprostol should be made available to all health services at all levels of care, and it is desirable that outpatient use be allowed, when indicated.

## Background

Misoprostol is a synthetic analogue of prostaglandin E1 (PGE1) with gastric secretion inhibitory and mucosal protection properties through the production of bicarbonate and mucus. It was first approved to be used to protect the stomach mucosa in patients using non-steroidal anti-inflammatory drugs. 1 This drug has been widely used in obstetric practice to induce abortion and as an agent to promote cervical ripening in induction of labor at term. The combination of misoprostol and mifepristone is used in medical abortions with a good safety profile in several countries. In Brazil, the commercialization of misoprostol is controlled for use in the hospital environment, in labor induction and legal abortion, or in cases of emptying of the uterus in abortion or retained dead fetus. There is widespread debate about the standardization of dosage in the use of misoprostol. Higher doses of misoprostol are used for induced and retained abortions, and much lower doses are used for cervical ripening and labor induction in term pregnancies. 2 It is also indicated for the treatment of postpartum hemorrhage (PPH).

## What are the pharmacokinetic properties of misoprostol?

Misoprostol is a synthetic analogue of PGE1. It is metabolized in the liver, deesterified and becomes the active metabolite, misoprostol acid. It has the ability to bind to uterine smooth muscle cells, increasing the strength and frequency of uterine contractions. 3 In the uterine cervix, it also promotes the breakdown of collagen in the connective tissue and a reduction in cervical tonus. 4 Misoprostol can be used orally, vaginally, sublingually and rectally. In oral administration, the drug reaches its maximum peak 20-30 minutes after ingestion, remaining detectable for up to four hours. Misoprostol administered sublingually is absorbed more quickly and has higher peak concentrations than when administered orally, which tends to cause higher rates of gastrointestinal side effects at any dose. 5 Overall bioavailability of the drug used vaginally is greater, since the absorption is slower than in other routes, and the maximum plasmatic peak is reached in 40-60 minutes, remaining stable up to two hours after application. The vaginal route also allows for greater effects on the cervix and uterus. 6 The pharmacokinetics of rectal misoprostol is similar to that of vaginal misoprostol, although with a lower overall bioavailability and a significantly lower peak plasma level. 7 It has been demonstrated that levels of misoprostol in breast milk are known to peak and decline rapidly with an average half-life of around one hour. Although it normally appears in colostrum and milk, the low levels detected suggest that a minimal amount of misoprostol could potentially be ingested by the newborn. 4

## What are the adverse effects and contraindications for using misoprostol?

Although other prostaglandins can cause myocardial infarction and bronchospasm, misoprostol is not associated with these effects. Toxic doses have not been well established and cumulative doses of up to 2,200 mcg in 12 hours are well tolerated without significant adverse effects. 8 A case of non-lethal misoprostol overdose was reported after ingestion of 6,000 mcg, coursing with hyperthermia, rhabdomyolysis, hypoxemia and metabolic acidosis. 9 One fatal case was reported after ingestion of 12,000 mcg (60 tablets), causing gastrointestinal bleeding with gastric and esophageal necrosis and organ failure. 10 The most common adverse effects of misoprostol are nausea, vomiting, diarrhea, abdominal pain, chills, shivering and fever. All these effects are dose-dependent. 8 Gastrointestinal effects may occur in approximately 35% of women and are more common after oral or sublingual administration. Diarrhea is the most common adverse effect and is usually mild and self-limited to one day. 11 Shivering and fever are also transitory effects and may occur in 28% and 7.5%, respectively, of women who used 600 mcg of misoprostol orally. 12 The occurrence of fever and shivering from misoprostol in the active management of the third stage favors the routine use of oxytocin as the drug of choice for the prevention of hemorrhage. 13 Although dose-dependent, uterine hyperstimulation is one of the most frequent adverse effects in labor induction. The risk of uterine hyperstimulation was high with high doses of misoprostol used in the past. With low doses (≤50 mcg of initial dose), the risk is similar to that of dinoprostone, 4-12%, depending on the route and dosage. 11 In a Cochrane meta-analysis, the risk of hyperstimulation with alteration of fetal heart rate was significantly lower with low-dose oral misoprostol (3.4%) compared to vaginal dinoprostone (7.0%; RR: 0.49; 0.40-0.59). In that same meta-analysis, a lower risk of hyperstimulation with fetal cardiac alteration was also found with oral misoprostol (3.9%), compared to the vaginal route (5.7%; RR: 0.69; 0.53-0.92). 14 Fetal distress, the presence of meconium in the amniotic fluid and uterine rupture may occur as a result of hyperstimulation (hypersystole or tachysystole with or without hypertonia). 15 Uterine rupture is the most feared adverse effect of labor induction, especially in women with previous uterine scar. Although extremely rare, there are case reports of uterine rupture during first-trimester abortion induction. Most cases of uterine rupture have been described in third-trimester inductions and associated with previous uterine scar or other risk factors. 7 The risk of uterine rupture in women with induction of labor for vaginal delivery after cesarean section with misoprostol is 6-12%. 11 Therefore, this is usually a contraindication for using the drug. 16 17 It is important to emphasize that misoprostol can be used in the second trimester in women with a previous cesarean section, since most studies point to a low risk of uterine rupture. 17 A meta-analysis identified that this risk is not significantly different when the woman has had a previous cesarean section (0.47%) compared to no uterine scar (0.08%; RR: 2.36; 0.39-14.32), although it is significantly higher with two or more previous cesarean sections (2.5%; RR: 17 ,55; 3-102.8). 18

## What are the teratogenic effects of misoprostol?

The Food and Drug Administration (FDA) classifies misoprostol as a category X drug (evidence of teratogenesis in animals and humans) in the first and second trimesters of pregnancy. Animal studies have shown a significant reduction in fertility with the use of high doses (6.25 to 625 times the maximum human therapeutic dose). In pregnant rabbits, doses of 300 to 1,500 mcg/kg of misoprostol on days 7-19 of embryogenesis have been associated with teratogenic effects. 19 Misoprostol-related malformations were initially described in case reports in humans. 20 21 22 These findings were subsequently confirmed in case-control and prospective studies and meta-analyses. 23 24 25 26 Most of these data come from Brazil and involve cases of malformations related to failed abortion with the use of misoprostol. In countries where abortion is legally permitted, patients rarely continue with the pregnancy after a failed abortion with misoprostol. In humans, there are several malformations associated with the use of misoprostol in the first trimester of pregnancy, such as: Moebius sequence (compromise of the VI and VII cranial nerves with paralysis of the eyes and facial muscles), arthrogryposis, transverse reduction of extremities and limbs, congenital clubfoot, hydrocephalus, encephalocele, meningocele, hemifacial microsomia, severe trismus. 21 26 27 The risk of any malformation associated with the use of misoprostol is 2.64 (95% confidence interval [CI]: 1.03-6.75) compared to the unexposed group, 25 while the risks for the Moebius sequence and transverse limb reduction were 25.31 (95% CI: 11.11-57.66) and 11.86 (95% CI: 4.86-28.90), respectively. 24 The teratogenic mechanism attributed to fetal malformations and alterations is a result of vascular disruption caused by intense uterine contractions and vaginal bleeding leading to embryonic hypoperfusion with tissue hypoxia, endothelial cell damage and tissue loss. 24 28 29 Fetal malformations and impairments depend on the developmental stage of the embryo, and the greatest risks are related to the use in the first trimester of pregnancy. It is still controversial if the risk of teratogenicity is dose-dependent, since studies indicate, for example, the association of severe malformations such as hydrocephalus with both low (200 mcg) and high doses (800 mcg) of misoprostol. 26 30 Hence, it is not possible to provide certainty regarding the absence or severity of alterations after using any dose of misoprostol in the first trimester of pregnancy.

## How to use misoprostol in uterine evacuation after embryonic death?

Misoprostol is used for uterine evacuation in first trimester pregnancy loss. On ultrasound examination, pregnancy loss can be characterized by the following aspects: presence of gestational sac without yolk sac or embryo and with mean diameter ≥ 25 mm; embryo with crown-rump length greater than or equal to 7 mm without cardiac activity; no embryo with a heartbeat two weeks after an examination demonstrating an empty gestational sac or no embryo with a heartbeat at 11 or more days after an examination demonstrating a gestational sac with yolk sac. In these situations, three approaches are possible: expectant management, mechanical uterine evacuation, or pharmacological evacuation. 31 The most effective and safe way to promote pharmacological uterine evacuation is the combination of mifepristone 200 mg followed by misoprostol (1-2 days later), with an efficacy rate of around 90% versus 70% when using misoprostol alone. 32 33 Given the unavailability of mifepristone, since its use is not regulated in Brazil, the isolated use of misoprostol is a reasonable alternative. There are several protocols, and the International Federation of Gynecology and Obstetrics (FIGO) 17 and the World Health Organization (WHO) 34 recommend the administration of 800 mcg vaginally, sublingually or buccally (four 200 mcg tablets). FIGO recommends a second dose three hours later. 17 34 There are no clear definitions regarding the interval and number of complementary doses, if necessary. Longer dosing intervals have the benefit of exposing the patient to a reduced risk of adverse effects. On the other hand, shorter dosing intervals (closer to three hours) may be necessary to generate sufficient uterine activity, particularly if misoprostol is given buccally or sublingually. Although uterine hyperstimulation is rare, particularly in the first trimester, the risk may increase with shorter dosing intervals. In pregnancies of less than 12 weeks, 1-3 doses of misoprostol are usually sufficient to expel the uterine contents. 35 The main advantages of using misoprostol include avoiding uterine perforation and formation of synechiae, reduced risks of sequelae inherent to the mechanical dilation of the cervix, and no need for anesthetic procedure. Disadvantages include a longer resolution time (sometimes days), higher prevalence of some symptoms such as cramps, bleeding, nausea, fever and chills, occasional need for surgical complementation and blood transfusion, and the woman's anxiety because of the waiting. 31 36 When opting for mechanical evacuation of the uterus, misoprostol can be used to prepare the cervix, avoiding or facilitating instrumental dilation before aspiration or curettage. The recommended dose is 400 mcg vaginally 3-4 hours before the procedure. If available, the sublingual route can be used in a shorter time interval (one hour). 17 31 Misoprostol 800 mcg vaginally (four 200 mcg tablets) is recommended for uterine evacuation in pregnancy loss up to 13 weeks.

## The use of misoprostol in legal abortion

Brazil has one of the most restrictive regulations related to induced abortion - induced abortion is only legally permitted in cases of pregnancy resulting from rape, risk to the woman's life and fetal anencephaly – and to the use of misoprostol in the world. In a study of countries in Africa, Asia and Latin America, Brazil was close only to Vietnam among those with greater restrictions on access to medical abortion in the world. 37 Brazil is the only South American country where misoprostol is not available directly to women, whether in health services or for sale in pharmacies. 38 Contrary to what one might imagine, these barriers fail to reduce the use of misoprostol by women, since half of illegal abortions in the country are performed with this drug. 39 The regimen of use of misoprostol alone recommended for the induction of abortion in cases provided for by law, is shown in chart 1 . 1 17 34 The drug is used until expulsion of products of conception. In the first trimester, three doses of misoprostol are usually sufficient to complete the treatment. 40

**Chart 1.** Regimen of use of misoprostol alone according to gestational age for uterine evacuation in induced abortion/legal abortion

Gestational age	Dosage
Up to 14 weeks	misoprostol 800 mcg (vaginally, sublingually, or buccally) every 3 hours
14- 24 weeks	misoprostol 400 mcg (vaginally, sublingually, or buccally) every 3 hours
25-28 weeks	misoprostol 200 mcg (vaginally, sublingually, or buccally) every 4 hours
Over 28 weeks	misoprostol 100 mcg every 6 hours

**Source:** Krugh M, Maani CV. Misoprostol. In: StatPearls [Internet]. Treasure Island: StatPearls Publishing; 2022 [cited 2022 Dec 15]. Available from: https://www.ncbi.nlm.nih.gov/books/NBK539873/ . Morris JL, Winikoff B, Dabash R, Weeks A, Faúndes A, Gemzell-Danielsson K, et al. FIGO's updated recommendations for misoprostol used alone in gynecology and obstetrics. Int J Gynaecol Obstet. 2017;138(3):363-6. doi: 10.1002/ijgo.12181. World Health Organization. Abortion care guideline [Internet]. Geneva: WHO; 2022 [cited 2022 Oct 31]. Available from: https://www.ncbi.nlm.nih.gov/books/NBK578942/pdf/Bookshelf_NBK578942.pdf . 1 17 34

In cervical preparation prior to surgical abortion in pregnancies over 12-14 weeks, the use of misoprostol 400 mcg (vaginally or orally) 2-3 hours before surgical treatment is routinely recommended. 34 If sublingual route is used, the time until the surgical procedure can be reduced to 1-2 hours. 40 Although cervical preparation should not be used routinely in pregnancies before 12 weeks, it can be beneficial in specific cases such as women at increased risk of complications during cervical dilation, for example, those with cervical anomalies or a history of cervical surgery. 34 41 Safety and efficacy data of the misoprostol treatment regimen alone for induced abortion were published in a randomized clinical trial of 2,066 women who received three doses of misoprostol 800 mcg. 42 In that study, only 0.04% of women had vaginal bleeding requiring return to the hospital. There were no serious adverse events among study participants. The WHO cites the possibility of the combined use of letrozole and misoprostol as safe and effective in terminating pregnancies of less than 13 weeks in scenarios where mifepristone is not available (letrozole 10 mg orally each day for three days followed by misoprostol 800 mcg sublingually on the fourth day). 34

## Is it safe to use misoprostol on an outpatient basis?

The use of misoprostol on an outpatient basis is considered effective and safe for the treatment of induced abortion, especially in the first 12 weeks of pregnancy. The use of misoprostol during this period has minimal adverse effects, such as diarrhea, vomiting, nausea and fever, which can be easily treated by professionals outside the hospital setting. 42 43 44 Outpatient use can reduce costs for both the health system and the hospital due to the waiver of hospitalization, as well as for women, since they do not need to remain in hospitals and in most cases, can receive adequate care at health units close to their homes. 34 In cases of induction of labor, the use of misoprostol in a hospital environment is recommended.

## How to induce emptying of the uterus with misoprostol in fetal death at 13-24 weeks?

When the diagnosis of fetal death is established, the health professional assisting this pregnant woman and her family must always be able to answer the posed questions with empathy and embracement, even if there are no answers to all. A systematic review including 14 controlled and randomized studies that evaluated the use of misoprostol in fetal death in the second and third trimesters found 100% effectiveness in uterine evacuation within 48 hours. 45 Randomized studies support the use of misoprostol as a first-line agent in the induction of labor in fetal death at 20-24 weeks, including in patients with a history of previous cesarean section. 46 47 Several intervals between doses, dosages and routes of administration are described, but none showed clear evidence of superiority. The regimen of misoprostol recommended for uterine evacuation in the case of fetal death at 14-24 weeks of gestational age is 400 mcg vaginally every 4-6 hours. 34

## How to use misoprostol in the induction of stillbirth after 24 weeks?

In cases of fetal death after more than 24 weeks, labor induction depends on the conditions of cervical maturation. In patients with a favorable cervix (Bishop index ≥ 6), labor induction can be started with oxytocin without the use of misoprostol for previous cervical ripening. In patients with an unfavorable cervix and without previous uterine scar, misoprostol is the agent of choice for preparing the cervix and inducing labor. 17 34 48 The following regimens are recommended:

- 25-26 weeks: misoprostol 400 mcg vaginally or sublingually every 4-6 hours;
- 27-28 weeks: misoprostol 100 mcg vaginally or sublingually every 4-6 hours;
- Over 28 weeks: misoprostol 25 mcg vaginally every six hours.

In patients with previous segmental scarring and unfavorable cervix at 24-28 weeks, cervical preparation can be performed with a mechanical method (transcervical balloon) followed by the use of oxytocin. The use of misoprostol seems to be an acceptable alternative at this gestational age, since the risk of uterine rupture is low. In a review study in which misoprostol was used at this gestational age, the risk of uterine rupture was 0.28% (95% CI: 0.08-1.00) in patients with a previous cesarean section versus 0.04% (95% CI: 0.01-0.20) in patients without a previous cesarean section. 18 49 50 However, at 24-26 weeks, low doses of misoprostol (100 mcg to 200 mcg per dose) may be suggested. 50 In pregnancies over 28 weeks, cervical preparation for labor induction should be performed in accordance with recommendations for parturient women with a live fetus.

## How to perform maturation of the uterine cervix and induction of labor with misoprostol?

In the labor induction process, when the situation of the uterine cervix is unfavorable, a maturation process is recommended to shorten the duration of induction and increase the chance of vaginal delivery. When the Bishop score is less than 6, the cervix is generally considered unfavorable, and mechanical and/or pharmacological methods can be used in this process. 16 51 Prostaglandins, including misoprostol, are contraindicated for cervical ripening or induction of labor in full-term pregnancies with previous cesarean section or other major uterine surgery due to the association with a higher risk of uterine rupture. 52 Pre-existing regular uterine activity is a relative contraindication to the use of misoprostol, as it can lead to excessive uterine activity. Delaying or avoiding administration should be considered if the patient has two or more painful contractions within 10 minutes, especially in patients who have already received at least one dose of prostaglandin. 53 In Brazil, misoprostol for vaginal use in labor induction is available in tablets containing 25 mcg of the drug. The 50 mcg dose is more effective than the 25 mcg dose, but leads to higher rates of tachysystole, cesarean delivery due to fetal compromise, admission to neonatal intensive care units, and meconium elimination. 16 54 The interval between doses can vary between 3-6 hours. The number of doses required for cervical maturation and/or effective labor varies. If necessary, oxytocin can be started four hours after the final dose of misoprostol. There are no definitions regarding the total limit of doses or the time of maturation and/or labor induction. 50 55 56 In some countries, a pessary with controlled release of misoprostol (200 mcg in 24 hours) is available. Comparative studies with the dinoprostone pessary have shown a significantly shorter mean time to vaginal delivery and a greater chance of tachysystole. 57 A 2021 meta-analysis supported the use of low doses of oral misoprostol for labor induction and suggested that an initial dose of 25 mcg can offer a good balance between efficacy and safety. 14 Other routes for the use of misoprostol in labor induction, including buccal and sublingual administration, have been less studied. Small trials suggest similar or inferior results to those of vaginal or oral administration. 58 59 60 In pregnancies over 26 weeks, the use of misoprostol at an initial dose of 25 mcg vaginally every 4-6 hours is recommended for cervical maturation prior to labor induction.

## How to use misoprostol to induce labor in women with a previous cesarean section?

Women planning a vaginal birth after a previous caesarean section (Trial of labor after cesarean – TOLAC) may need labor induction. There are two concerns: reduced chances of vaginal birth after caesarean section (VBAC) and increased risk of uterine rupture. Having a previous vaginal delivery and a favorable cervix are the main predictors of induction resulting in VBAC. 61 Induction itself does not reduce the chances of VBAC when compared with expectant management. 62 The major risk is uterine rupture related to induction. Regardless of the method used for induction, women with a previous cesarean section and induced labor are at greater risk of uterine rupture than those in labor with spontaneous delivery or expectant management. The frequency of uterine rupture in women at full-term who had labor induced was almost twice as high as the frequency in women in whom labor began spontaneously (1.5% versus 0.8%). 63 The factors associated with an increased risk of rupture during induced TOLAC include:

- No previous vaginal delivery – for example, in a study, the risks of rupture during TOLAC-induced in women without a previous vaginal delivery versus a previous vaginal delivery were 1.5% and 0.6%, respectively; 61 64
- Use of prostaglandins – induction with prostaglandins appears to be associated with a greater risk of uterine rupture than induction with oxytocin or cervical ripening with mechanical methods followed by administration of oxytocin. 64

*Risk of rupture with prostaglandin use* – Data from large randomized trials and from good quality observational studies on the effects of prostaglandins alone or in combination with other agents for cervical ripening in TOLAC are not available. Much data on prostaglandin use in women with a previous caesarean section has been derived from observational studies in which misoprostol (PGE1) was used. Reports on the use of other prostaglandins, such as prostaglandin E2, are limited by their small size, the co-administration of other agents and the lack of stratification by previous vaginal delivery. 65 *Unspecified prostaglandin* – Concern over the use of prostaglandins arose following the publication of a large population-based retrospective cohort study that analyzed data from 20,095 primiparous women who delivered after a single previous cesarean section. 65 In that study, the rate of uterine rupture was similar for women in spontaneous labor and those induced without the use of prostaglandin, but significantly higher among women induced with prostaglandin (type not available). The specific uterine rupture rate by category was:

- Repeat cesarean sections without labor: 1.6 ruptures per 1,000 planned repeat cesareans;
- Spontaneous labor: 5.2 ruptures per 1,000 spontaneous deliveries;
- Induced labor (without prostaglandins): 7.7 ruptures per 1,000 labors induced without the use of prostaglandins;
- Induced labor (with prostaglandins): 24.5 ruptures per 1,000 labors induced using prostaglandins. Compared to repeat cesarean delivery, the relative risk of rupture with the use of prostaglandins was 15.6 (95% CI: 8.1-30.0).

However, despite the very large number of cases, the information in this study is from a database and individual reviews of medical records were not performed to check other medications administered. The risk of uterine rupture reported in this retrospective study was lower in another large prospective study. 66 In that study, the rate of uterine rupture among patients induced with prostaglandin with or without oxytocin was lower – 14 per 1,000 induced deliveries –, although still considerably high. Specifically on misoprostol (PGE1), a randomized trial on the use of misoprostol for cervical ripening in labor induction in women with previous cesarean sections was stopped early because of safety concerns due to uterine rupture. 67 68 This study and several case reports have led some researchers to conclude that misoprostol is associated with a greater risk of uterine rupture than other prostaglandins and therefore should not be used in women planning a TOLAC. 51 68 69 70 71 The positions of Gynecology and Obstetrics Societies worldwide are:

- American College of Obstetricians and Gynecologists (ACOG –United States) 64 – advises that misoprostol should not be used for cervical ripening or labor induction in women at term with any previous uterine incision and does not address the use of prostaglandin E2;
- Society of Obstetricians and Gynecologists of Canada (SOGC – Canada) 72 – has the same position regarding the use of misoprostol, but allows the use of prostaglandin E2 (dinoprostone) in some circumstances and after appropriate advice;
- National Institute for Health and Care Excellence (United Kingdom) 51 – concluded that if childbirth is indicated, women who have had a previous cesarean section can receive labor induction with vaginal prostaglandin E2, but do not mention misoprostol. 51 73

In conclusion, the use of misoprostol in women with previous cesarean is not recommended given the higher risk of uterine rupture. Note that mechanical methods are available, effective and safe.

## How to use misoprostol to induce labor in ruptured membranes?

Premature rupture of membranes (PROM) is one of the most common complications of term and preterm pregnancies, but there is a gap in knowledge about how management affects the cesarean rate. As gestational age at delivery is the critical factor influencing perinatal outcome, expectant management is generally adopted when far from term. In PROM at term, the risk of maternal and fetal infectious morbidity increases with longer duration of membrane rupture. Therefore, expectant management should be brief, with instructions for induction of labor. 74 Meta-analyses conclude that misoprostol is an effective and safe agent for inducing labor in women with PROM at term. Compared to oxytocin, the risk of contraction abnormalities and the rate of maternal and neonatal complications were similar between the two groups. 74 75 Misoprostol 25 mcg should be considered as the starting dose for cervical ripening and labor induction in women with PROM. The frequency of administration should not exceed 3-6 hours. Furthermore, oxytocin should not be administered less than 4 hours after the last dose of misoprostol. Misoprostol at higher doses (50 mcg every six hours) may be appropriate in some situations, although higher doses may be associated with an increased risk of complications, including uterine tachysystole with fetal heart rate decelerations. 16 A Cochrane Review suggests the immediate induction of labor in patients with PROM at term. Compared with expectant management, induction of labor is associated with a reduction in maternal and possibly neonatal infection and lower treatment costs, without an increase in cesarean sections. 76 In conclusion, the use of misoprostol is recommended as a safe and effective option for women with PROM and unfavorable cervix, provided they do not have contraindications for the use of this medication, such as, for example, previous cesarean section.

## Misoprostol in the management of postpartum hemorrhage: how to use it?

Postpartum hemorrhage affects around 2% of all patients, and in only 25% of cases the risk factors are pronounced. The obstetrician must perform prophylaxis in 100% of cases and be aware of the occurrence of PPH, even if drug prophylaxis is performed. There is strong evidence that the association of uterotonics prescribed in the immediate postoperative period of childbirth reduces blood loss greater than 500 mL: ergometrine plus oxytocin (RR: 0.70; 95% CI: 0.59-0.84) and misoprostol plus oxytocin (RR: 0.70; 95% CI: 0.58-0.86) and reduces the need for blood products (RR: 0.51; 95% CI: 0.37-0.70). 77 This is not only a result of the combination of the strength of the two drugs, but also because oxytocin is thermolabile and it is difficult to guarantee a cold chain throughout the medication production, transportation and dispensing route. However, the association of two uterotonics increases the occurrence of side events, mainly vomiting (RR: 2.11; 95% CI: 1.39-3.18). Therefore, the use of two uterotonics is recommended for patients at high risk of PPH, always bearing in mind the contraindication of ergometrine for hypertensive/pre-eclampsia patients. The following uterotonics are recommended for the prophylaxis of PPH:

Oxytocin:

- In post-vaginal delivery: single dose of 10 IU intramuscularly right after birth;
- In cesarean section: 5 IU in slow intravenous infusion in three minutes and maintenance solution (20 IU of oxytocin in 500 ml of 0.9% saline solution intravenously at 125 ml/h for 4-12 hours);
- Misoprostol: single dose of 600 mcg rectally;
- Ergometrine: single dose of 0.2 mg intramuscularly.

For the drug treatment of PPH, the use of misoprostol 800 mcg rectally is recommended. It is important to remember that since the onset of action of rectal misoprostol is slower than that of other uterotonics, it should be used as an adjuvant to treatment with oxytocin. Misoprostol should not be used in isolation, maintaining uterine massage until the onset of its effect, which may take 15-20 minutes. Always consider the use of tranexamic acid 1 g intravenously over 10 minutes, with the possibility of repeating the 1 g dose in 30 minutes if bleeding persists. 78

## What regulations are related to the use of misoprostol?

Circular letter number 182/2021 of the Office of the President of the Brazilian Federal Council of Medicine, 79 expressed the impossibility of using misoprostol outside the hospital setting. The letter highlights the Ordinance of the Brazilian National Health Surveillance Agency (Anvisa) number 344/98, 80 of the Secretariat for Health Surveillance of the Ministry of Health, according to which misoprostol is on list C1 that includes substances subject to special control (prescription in two copies), with the addendum that the purchase and use of medication containing the substance misoprostol will only be allowed in hospitals duly registered with the Sanitary Authority. In its guide, the WHO (World Health Organization, 2018) 81 recognizes that the home use of misoprostol is a safe and effective option for women. In addition, the drug was added to the WHO list of essential drugs in 2019, at the same time that the need for in-person medical supervision to administer pharmacological abortion was withdrawn. In Brazil, Anvisa ordinances and resolutions and manifestations of the Federal Council of Medicine currently establish that misoprostol has exclusive hospital use with special control. Compared to other countries in the world and to WHO recommendations, there is excessive difficulty in accessing and releasing the use of misoprostol in Brazil. Given the existence of a robust body of evidence, there are no scientific justifications for imposing other restrictions on misoprostol, in addition to those related to special control drugs, i.e. prescription in two copies with retention of one copy in the pharmacy, and the possibility of identifying who prescribed the induced abortion treatment.

## Final considerations

In obstetric practice, misoprostol has been widely used in legal abortion, uterine emptying due to embryonic or fetal death, cervical ripening and labor induction, and management of PPH. Contrary to the accumulated scientific evidence, Brazil has one of the most restrictive regulations in the world related to the use of misoprostol. The great difficulty in acquiring, storing and dispensing the medication imposed by Ordinance No. 344/1998 of Anvisa, still in force, contributes to denying the right to safer outpatient treatments for women who need it. These restrictions also hinder the availability of this medication, essential and mandatory, in obstetric care services.

National Commission Specialized in Childbirth, Puerperium and Abortion Care of the Brazilian Federation of Gynecology and Obstetrics Associations (Febrasgo)

President:

Alberto Trapani Júnior

Vice-President:

Alessandra Cristina Marcolin

Secretary

Sheila Koettker Silveira

Membros:

Elias Ferreira de Melo Junior

Liduina de Albuquerque Rocha e Sousa

Marcia Maria Auxiliadora de Aquino

Mirela Foresti Jiménez

Ricardo Porto Tedesco

Tenilson Amaral Oliveira

National Commission Specialized in Antenatal Care of the Brazilian Federation of Gynecology and Obstetrics Associations (Febrasgo)

President:

Fernanda Garanhani de Castro Surita

Vice-President:

Lílian de Paiva Rodrigues Hsu

Secretary

Adriana Gomes Luz

Membros:

Jorge Oliveira Vaz

Eliana Martorano Amaral

Eugenia Glaucy Moura Ferreira

Francisco Herlanio Costa Carvalho

Joeline Maria Cleto Cerqueira

Jose Meirelles Filho

Luciana Silva dos Anjos França

Marianna Facchinetti Brock

Mary Uchiyama Nakamura

Patricia Goncalves Teixeira

Renato Ajeje

Sergio Hecker Luz

National Commission Specialized in Gestational Trophoblastic Disease of the Brazilian Federation of Gynecology and Obstetrics Associations (Febrasgo)

President:

Antonio Rodrigues Braga Neto

Vice-President:

José Mauro Madi

Secretário

Mauricio Guilherme Campos Viggiano

Membros:

Bruno Maurizio Grillo

Christiani Bisinoto de Sousa

Claudio Sergio Medeiros Paiva

Elaine Azevedo Soares Leal

Elza Maria Hartmann Uberti

Fabiana Rebelo Pereira Costa

Izildinha Maesta

Jose Arimatea dos Santos Junior

Maria do Carmo Lopes de Melo

Rita de Cassia Alves Ferreira Silva

Sue Yazaki Sun

Tiago Pedromonico Arrym

National Commission Specialized in High Risk Pregnancy of the Brazilian Federation of Gynecology and Obstetrics Associations (Febrasgo)

President:

Rosiane Mattar

Vice-President:

Alberto Carlos Moreno Zaconeta

Secretary

Mylene Martins Lavado

Membros:

Arlley Cleverson Belo da Silva

Carlos Alberto Maganha

Elton Carlos Ferreira

Felipe Favorette Campanharo

Inessa Beraldo de Andrade Bonomi

Janete Vettorazzi

Maria Rita de Figueiredo Lemos Bortolotto

Fernanda Santos Grossi

Renato Teixeira Souza

Sara Toassa Gomes Solha

Vera Therezinha Medeiros Borges

National Commission Specialized in Fetal Medicine of the Brazilian Federation of Gynecology and Obstetrics Associations (Febrasgo)

President:

Mario Henrique Burlacchini de Carvalho

Vice-President:

José Antonio de Azevedo Magalhães

Secretary

Roseli Mieko Yamamoto Nomura

Membros:

Alberto Borges Peixoto

Carlos Henrique Mascarenhas Silva

Carolina Leite Drummond

Edward Araujo Júnior

Fernando Artur Carvalho Bastos

Guilherme Loureiro Fernandes

Jair Roberto da Silva Braga

Jorge Fonte de Rezende Filho

Marcello Braga Viggiano

Maria de Lourdes Brizot

Nadia Stella Viegas dos Reis

Reginaldo Antônio de Oliveira Freitas Júnior

Rodrigo Ruano

National Commission Specialized in Maternal Mortality of the Brazilian Federation of Gynecology and Obstetrics Associations (Febrasgo)

President:

Marcos Nakamura Pereira

Vice-President:

Rodolfo de Carvalho Pacagnella

Secretary

Melania Maria Ramos de Amorim

Membros:

Acacia Maria Lourenço Francisco Nasr

Douglas Bernal Tiago

Elvira Maria Mafaldo Soares

Fatima Cristina Cunha Penso

Ida Perea Monteiro

João Paulo Dias de Souza

Lucila Nagata

Maria do Carmo Leal

Monica Almeida Neri

Monica Iassanã dos Reis

Jacinta Pereira Matias

Penha Maria Mendes da Rocha

National Commission Specialized in Obstetric Emergencies of the Brazilian Federation of Gynecology and Obstetrics Associations (Febrasgo)

President:

Alvaro Luiz Lage Alves

Vice-President:

Gabriel Costa Osanan

Secretary

Samira El Maerrawi Tebecherane Haddad

Membros:

Adriana Amorim Francisco

Alexandre Massao Nozaki

Brena Carvalho Pinto de Melo

Breno José Acauan Filho

Carla Betina Andreucci Polido

Eduardo Cordioli

Frederico Jose Amedee Peret

Gilberto Nagahama

Laises Braga Vieira

Lucas Barbosa da Silva

Marcelo Guimarães Rodrigues

Rodrigo Dias Nunes

Roxana Knobel

National Commission Specialized in Sexual Violence and Pregnancy Interruption Provided for by Law of the Brazilian Federation of Gynecology and Obstetrics Associations (Febrasgo)

President:

Robinson Dias de Medeiros

Vice-President:

Cristião Fernando Rosas

Secretary

Helena Borges Martins da Silva Paro

Membros:

Aline Veras Morais Brilhante

Anibal Eusébio Faúndes Latham

Débora Fernandes Britto

Edison Luiz Almeida Tizzot

Isabelle Cantidio Fernandes Diogenes

Kenia Zimmerer Vieira

Michele Lopes Pedrosa

Osmar Ribeiro Colas

Rivaldo Mendes de Albuquerque

Rosires Pereira de Andrade

Suely de Souza Resende

Zelia Maria Campos

National Commission Specialized in Professional Defense and Appreciation of the Brazilian Federation of Gynecology and Obstetrics Associations (Febrasgo)

President:

Maria Celeste Osorio Wender

Membros:

Carlos Henrique Mascarenhas Silva

Etelvino de Souza Trindade

Henrique Zacharias Borges Filho

Juvenal Barreto Borriello de Andrade

Lia Cruz Vaz da Costa Damásio

Maria Rita de Souza Mesquita

Mirela Foresti Jiménez

Sergio Hofmeister de Almeida Martins Costa

Celia Regina da Silva

Aljerry Dias do Rego

Rosires Pereira de Andrade

Maria Auxiliadora Budib

Carlos Alberto Sa Marques

Hilka Flavia Barra do Espirito Santo Alves Pereira
